# High colonization rates of extended-spectrum β-lactamase (ESBL)-producing *Escherichia coli*in Swiss Travellers to South Asia– a prospective observational multicentre cohort study looking at epidemiology, microbiology and risk factors

**DOI:** 10.1186/1471-2334-14-528

**Published:** 2014-10-01

**Authors:** Esther Kuenzli, Veronika K Jaeger, Reno Frei, Andreas Neumayr, Susan DeCrom, Sabine Haller, Johannes Blum, Andreas F Widmer, Hansjakob Furrer, Manuel Battegay, Andrea Endimiani, Christoph Hatz

**Affiliations:** Division for Infectious Diseases and Hospital Epidemiology, University Hospital Basel, Basel, Switzerland; Swiss Tropical and Public Health Institute, Basel, Switzerland; Department of Rheumatology, University Hospital Basel, Basel, Switzerland; Division of Clinical Microbiology, University Hospital Basel, Basel, Switzerland; Division of Communicable Diseases, Institute for Social and Preventive Medicine, University of Zurich, Zurich, Switzerland; Department of Infectious Diseases, Bern University Hospital and University of Bern, Bern, Switzerland; Institute for Infectious Diseases, University of Bern, Bern, Switzerland

**Keywords:** Colonization, *Enterobacteriaceae*, ESBL, Traveller, South Asia

## Abstract

**Background:**

International travel contributes to the worldwide spread of multidrug resistant Gram-negative bacteria. Rates of travel-related faecal colonization with extended-spectrum β-lactamase (ESBL)-producing *Enterobacteriaceae* vary for different destinations. Especially travellers returning from the Indian subcontinent show high colonization rates. So far, nothing is known about region-specific risk factors for becoming colonized.

**Methods:**

An observational prospective multicentre cohort study investigated travellers to South Asia. Before and after travelling, rectal swabs were screened for third-generation cephalosporin- and carbapenem-resistant *Enterobacteriaceae*. Participants completed questionnaires to identify risk factors for becoming colonized. Covariates were assessed univariately, followed by a multivariate regression.

**Results:**

Hundred and seventy persons were enrolled, the largest data set on travellers to the Indian subcontinent so far. The acquired colonization rate with ESBL-producing *Escherichia coli* overall was 69.4% (95% CI 62.1-75.9%), being highest in travellers returning from India (86.8%; 95% CI 78.5-95.0%) and lowest in travellers returning from Sri Lanka (34.7%; 95% CI 22.9-48.7%). Associated risk factors were travel destination, length of stay, visiting friends and relatives, and eating ice cream and pastry.

**Conclusions:**

High colonization rates with ESBL-producing *Enterobacteriaceae* were found in travellers returning from South Asia. Though risk factors were identified, a more common source, i.e. environmental, appears to better explain the high colonization rates.

**Electronic supplementary material:**

The online version of this article (doi:10.1186/1471-2334-14-528) contains supplementary material, which is available to authorized users.

## Background

According to the Global Report on Surveillance of antimicrobial resistance of the World Health Organization (WHO), resistance rates to third-generation cephalosporins of up to 50-80% have been described for all WHO regions. This increase in multidrug-resistance is not only associated with increased morbidity and mortality and higher cost, but with the need to use broader spectrum antibiotics to treat common infections, therefore further facilitating resistance development [1, 2].

Originally, multidrug-resistant (MDR) Gram-negative bacteria were considered to occur mainly in healthcare settings. However, since the late 1990s, the importance of ESBL-producing *Escherichia coli* (ESBL-*Ec*) as a major cause for community-acquired infections has become evident. Recent analyses showed that up to 70% of infections due to ESBL-*Ec* are community-acquired [3, 4].

The reasons for this increase in infections caused by ESBL-*Ec* are manifold, including: the ”classical“ risk factors such as previous antibiotic use (cephalosporins and fluoroquinolones), age, and the presence of co-morbidities [2]. Moreover, animals, food, and the environment serve as reservoirs for MDR Gram-negative bacteria [5]. While the evidence for direct animal-to-human transmission of MDR bacteria or their respective mobile genetic resistance traits is mainly circumstantial [5], more robust evidence for transmission via the food chain exists [6, 7]. Furthermore, investigations in recent years have shown international travel as a possible means of spreading ESBL-producing *Enterobacteriaceae* (ESBL-*Ent*), mainly ESBL-*Ec*, from high- to low-prevalence countries through asymptomatic travellers [8–13], thereby increasing the local prevalence and changing the local epidemiology with the import of non-autochthonous ESBL-*Ent*[14].

The impact of travelling on the local epidemiology of ESBL-*Ent* might be considerable, due to intense international travel [15] and very high colonization rates associated with some travel destinations [8, 9, 11–13]. An even greater threat, though occurring to a much lesser extent so far, is the spread of carbapenemase-producing *Enterobacteriaceae* (Carb-*Ent)*, such as NDM and OXA-48-like-producers [16, 17].

Several studies have tried to identify risk factors for becoming colonized while travelling [8, 9, 11–13, 18–20]. However, besides region visited, and, to some extent, suffering from gastrointestinal symptoms while travelling, the findings varied between the studies. As all these surveys looked at multiple travel destinations, this could be explained by risk factors varying among different regions of the world. Otherwise, especially in highly-endemic areas (like India) [21], ESBL-*Ec* could occur in such abundance in the environment that their acquisition no longer depends on singular risk factors.

The primary aim of this study was to investigate colonization rates in travellers returning from South Asia, a travel destination known to be associated with high colonization rates [8, 9, 11–13]. By focusing on one region of the world, the question of region-dependent risk factors for becoming colonized could be addressed. Moreover, more information on the regional distribution of different *E. coli* isolates was collected. Most importantly, the knowledge about the mechanism behind becoming colonized with ESBL-*Ec* in South Asia may be extrapolated to Carb-*Ent*, thereby helping to contain their spread.

## Methods

### Study population

Between December 2012 and October 2013, healthy travellers who planned to travel to South Asia (India, Bhutan, Nepal and Sri Lanka) and received pre-travel advice at the two largest Travel Clinics of German-speaking Switzerland, were enrolled in the study. There was no age restriction for inclusion in the study. Clients travelling for more than five weeks, travelling to other countries than India, Bhutan, Nepal, and Sri Lanka or to more than one of these countries, were excluded from participation. The study was approved by the local ethics committees (Ethikkommission beider Basel (Nr. 239/12), Kantonale Ethikkommission Zürich (Nr. 2013–0066). All participants gave written informed consent. A parent or a legal guardian had to sign the informed consent on behalf of minors.

### Collection of samples

Faecal samples were collected through self-applied rectal swabs (M40 Transystem, Copan Italia, Brescia, Italy) in the week before and directly after travelling and then sent by mail to the Division of Clinical Microbiology, University Hospital Basel. Oral and written explanations of how to use the swabs were provided for all participants. On each occasion, a questionnaire assessing risk factors for becoming or remaining colonized was completed. The questionnaire was paper-based and consisted of multiple choice or one-word answers.

### Microbiological analysis

Faecal samples were cultured overnight at 37°C in trypticase soy broth with 0.5% sodium chloride [22], followed by inoculation of a selective chromogenic medium for the detection of third-generation cephalosporin resistant organisms (chromID® ESBL, bioMérieux, Marcy-l’Etoile, France) and a MacConkey agar (Becton Dickinson, Breda, The Netherlands) with an ertapenem disk. Colonies growing on the chromogenic agar plate and within the ertapenem disk zone were identified using Vitek-2® system (bioMérieux, Hazelwood, Mo, USA). The Vitek-2® system was also used for susceptibility testing; results were interpreted according to the European Committee on Antimicrobial Susceptibility Testing (EUCAST) [23]. Cefoxitin was used to screen for acquired AmpC-production. Minimum inhibitory concentration (MIC) values for several antibiotics including meropenem and ertapenem were determined by Etest® (bioMérieux). To phenotypically detect ESBL, AmpC and carbapenemase production, we performed various combination disk tests (ROSCO Neosensitabs, Rosco Diagnostica, Taastrup Denkmark) and the modified Hodge test according to Clinical and Laboratory Standards Institute (CLSI) guidelines. The ROSCO Neo-Sensitabs were used according to the manufacturer’s recommendations and Hansen et al. [24].

### Molecular characterization of isolates

Eighty randomly selected *E. coli* isolates resistant to third-generation cephalosporins or those possessing a phenotype suggestive of ESBL production were screened using the CT-104 microarray (Check-Points, PD Wageningen, The Netherlands) to screen for the presence of class A (*bla*_TEM_, *bla*_SHV_, *bla*_CTX-M_*, bla*_KPC_), class B (*bla*_NDM_, *bla*_VIM_, *bla*_IMP_), class C plasmid-mediated AmpCs (pAmpCs) (*bla*_CMY_, *bla*_FOX_, *bla*_DHA_, *bla*_MOX_, *bla*_ACC_, *bla*_ACT_), and class D (*bla*_OXA-48-like_) β-lactamase genes. Genomic extraction was performed using the QIAmp DNA mini kit (QIAGEN). PCR/DNA sequence analyses for the *bla* genes (i.e., *bla*_SHV_ and *bla*_CTX-M_) found with the microarray were performed using primers and conditions previously reported [4, 17]. Sequences were analysed using DNAStar (Lasergene), translated into protein sequences (ExPASy; http://ch.expasy.org/), and compared to those previously described (http://www.lahey.org/Studies).

### Analysis of clonal relatedness

The relatedness of the 80 representative *E. coli* isolates was analysed by repetitive extragenic palindromic polymerase chain reaction (rep-PCR) as previously described. Isolates were defined as clonally-related when they had >85% genetic relatedness [25]. Multi locus sequence typing (MLST) was also performed for a randomly selected subgroup of 34 isolates according to the Achtman scheme (http://mlst.warwick.ac.uk/mlst/dbs/Ecoli) to obtain the specific sequence type (ST).

### Statistical analysis

Data were analysed using Stata 11 (StataCorp LP, Texas, USA). Univariate analysis was performed, and differences in proportions of categorical variables were compared using Pearson‘s χ^2^-test. The normality of continuous variables was assessed and the mean/median was reported and compared using Student’s t-test/Wilcoxon-Mann–Whitney test as appropriate. 95% confidence intervals (CI) for colonization rates were calculated using the Wilson score. A stepwise backward multivariate logistic regression was carried out to identify potential risk factors for colonization. Variables identified in the univariate analysis to be significantly associated with becoming colonized as well as variables with a p-value <0.1 in the stepwise backward approach were included in the final regression model.

## Results

Overall, 226 participants were enrolled, of whom 190 provided a pre- and post-travel faecal sample. Of these, eleven participants were excluded because they travelled to more than one of the above-mentioned countries. Of the remaining 179 participants, 170 (95.0%) had a negative pre-travel screening for ESBL-*Ent*, five (2.8%) had a positive screening and pre-travel data was missing for four participants (2.2%). Thus, the analysis is based on the data from 170 participants with a negative pre-travel screening.

Overall, 68 travellers went to India, 49 to Sri Lanka, 39 to Nepal and 14 to Bhutan. Median age was 41 years (range: 1–73 years), mean length of stay was 18.4 days (range: 5–35 days, standard deviation (SD): 6.5 days). Seventy-five travellers (44.1%) were male. Only 7 travellers (4.6%) took antibiotics while travelling (missing data regarding antibiotic use for 16 travellers, 12 of whom became colonized). Of these, 5 (71.4%) became colonized compared to 101 (68.7%) travellers not using any antibiotics (p = 0.879). The baseline characteristics and demographic data are shown in Table 1.

**Table 1:** **Demographic data and travel associated risk factors analysed**

		Total^a^	Neg-to-Pos^a,b^	Neg-to-Neg^a,c^	p-value^d^
Travellers		170 (100.0)	118 (69.4)	52 (30.6)	
Destination	India	68 (40.0)	59 (86.8)	9 (13.2)	<0.001
	Bhutan	14 (8.2)	11 (78.6)	3 (21.4)	
	Nepal	39 (22.9)	31 (79.5)	8 (20.5)	
	Sri Lanka	49 (28.9)	17 (34.7)	32 (65.3)	
Age (yrs, median, IQR)		41 (30–53)	41 (30–53)	40 (29–52)	0.899
Sex	Female	95 (55.9)	66 (55.9)	29 (55.8)	0.984
	Male	75 (44.1)	52 (44.1)	23 (44.2)	
Length of stay	< 2 weeks	34 (20.0)	21 (61.8)	13 (38.2)	0.548
	2-3 weeks	75 (44.1)	53 (70.7)	22 (29.3)	
	> 3 weeks	61 (35.9)	44 (72.1)	17 (27.9)	
Weight (kg, median, IQR)		70 (58–78)	70 (59–78)	70 (57–78)	0.685
Travel Reason	Tourist	115 (67.7)	73 (61.9)	42 (80.8)	0.047
	Business	23 (13.5)	18 (15.3)	5 (9.6)	
	VFR	32 (18.8)	27 (22.9)	5 (9.6)	
Sleeping Place	Hotel	81 (48.2)	56 (48.3)	25 (48.1)	0.298
	Guest House	62 (36.9)	40 (34.5)	22 (42.3)	
	Private Household	17 (10.1)	15 (12.9)	2 (3.9)	
	Other	8 (4.8)	5 (4.3)	3 (5.7)	
Eating Place	Restaurant	135 (81.3)	94 (81.7)	41 (80.4)	0.837
	Private	31 (18.7)	21 (18.3)	10 (19.6)	
Daily Alcohol	No	126 (74.1)	90 (76.3)	36 (69.2)	0.334
	Yes	44 (25.9)	28 (23.7)	16 (30.8)	
Tap Water Consumption	No	101 (59.4)	77 (65.2)	24 (46.2)	0.019
	Yes	69 (40.6)	41 (34.8)	28 (53.8)	
Dairy Products	No	21 (12.3)	11 (9.3)	10 (19.2)	0.070
	Yes	149 (87.7)	107 (90.7)	42 (80.8)	
Fruits	No	144 (85.7)	99 (84.6)	45 (88.2)	0.538
	Yes	24 (14.3)	18 (15.4)	6 (11.8)	
Salad	No	70 (41.4)	53 (45.3)	17 (32.7)	0.125
	Yes	99 (58.6)	64 (54.7)	35 (67.3)	
Ice Cream and Pastry	No	78 (45.9)	48 (40.7)	30 (57.7)	0.040
	Yes	92 (54.1)	70 (59.3)	22 (42.3)	
Meat	No	33 (19.4)	27 (81.8)	6 (18.2)	0.085
	Yes	137 (80.6)	91 (66.4)	46 (33.6)	
Travellers’ Diarrhoea	No	106 (62.7)	70 (59.3)	36 (70.6)	0.164
	Yes	63 (37.3)	48 (40.7)	15 (29.4)	
PPI	No	164 (96.5)	114 (96.6)	50 (96.1)	0.882
	Yes	6 (3.5)	4 (3.4)	2 (3.9)	

**Note.** IQR, interquartile range; PPI, proton pump inhibitor; VFR, visiting friends and relatives.

^**a**^Data are presented as numbers and (**%**) if not otherwise indicated.

^**b**^Neg-to-Pos, people non-colonized before the trip and colonized with ESBL-*Ent* immediately after they return.

^**c**^Neg-to-Neg, people non-colonized before and after the trip to South Asia.

^d^Derived by Pearson’s χ^2^-test or Student’s t-test/Wilcoxon-Mann–Whitney test.

After return, the overall colonization rate with ESBL-*Ent* was 69.4% (95% CI 62.1-75.9%). The highest rate was found in travellers returning from India (86.8%; 95% CI 78.5-95.0%) and the lowest in travellers returning from Sri Lanka (34.7%; 95% CI 22.9-48.7%). Rates for Nepal and Bhutan were 79.5% (95% CI 64.5-89.2%) and 78.6% (95% CI 52.4-92.4%), respectively. The colonization rates for India, Nepal, and Bhutan were significantly higher than the one for Sri Lanka (p < 0.001). ESBL-*Ec* were found in all of the colonized travellers, but three had an additional ESBL-producing *Klebsiella pneumoniae* and one an ESBL-producing *Citrobacter freundii*. One traveller returning from India was colonized with a carbapenemase-producing *E. coli* (confirmed by PCR and sequencing as New Delhi metallo-beta-lactamase-1, NDM-1).

The main risk factors for becoming colonized identified in the univariate analysis, besides travel destination, were reason for travelling (visiting friends and relatives as compared to tourists) and consumption of ice cream and pastry (Table 1).

In the multivariate logistic regression analysis, travelling to India, Bhutan, or Nepal (as compared to travelling to Sri Lanka), visiting friends and relatives (as compared to travelling as a tourist), consumption of ice cream and pastry, as well as length of stay showed a significant association with becoming colonized with ESBL-*Ent*. Drinking tap water was associated with a decreased risk (Table 2).

**Table 2:** **Travel-associated risk factors for colonization: univariate and multivariate logistic regression model**

		OR (95% CI)	p-value^a^	adjusted OR^b^(95% CI)	p-value^a^
Destination	India	1		1	
	Bhutan	0.56 (0.13-2.40)	0.434	0.66 (0.13-3.30)	0.615
	Nepal	0.59 (0.21-1.68)	0.325	0.57 (0.17-1.88)	0.355
	Sri Lanka	0.08 (0.03-0.20)	< 0.001	0.05 (0.02-0.16)	< 0.001
Age		1.00 (0.98-1.02)	0.722		
Sex	Female	1.00			
	Male	0.99 (0.52-1.92)	0.984		
Length of Stay (per week)		1.26 (0.88-1.80)	0.215	2.08	0.010
Weight		1.01 (0.99-1.03)	0.416		
Travel Reason	Tourist	1		1	
	Business	2.07 (0.72-5.98)	0.179	1.58 (0.44-5.71)	0.483
	VFR	3.11 (1.11-8.68)	0.031	3.86 (1.02-14.59)	0.046
Sleeping Place	Hotel	1			
	Guest House	0.81 (0.40-1.64)	0.560		
	Private Household	3.35 (0.71-15.76)	0.126		
	Other	0.74 (0.16-3.36)	0.701		
Eating Place	Restaurant	1			
	Private	0.92 (0.40-2.12)	0.837		
Daily Alcohol	No	1			
	Yes	0.70 (0.34-1.45)	0.335		
Tap Water Consumption	No	1		1	
	Yes	0.52 (0.21-1.28)	0.154	0.27 (0.08-0.87)	0.029
Dairy Products	No	1			
	Yes	2.32 (0.92-5.86)	0.076		
Fruits	No	1			
	Yes	1.36 (0.51-3.67)	0.539		
Salad	No	1			
	Yes	0.59 (0.30-1.16)	0.126		
Ice Cream and Pastry	No	1		1	
	Yes	1.99 (1.03-3.85)	0.042	3.90 (1.61-9.43)	0.002
Meat	No	1			
	Yes	0.44 (0.17-1.14)	0.091		
Travellers’ Diarrhoea	No	1			
	Yes	1.65 (0.81-3.33)	0.166		
PPI	No	1			
	Yes	0.88 (0.16-4.95)	0.880		

**Note:** OR, odds ratio; CI, confidence interval.

^a^derived by Wald test.

^b^adjusted for the other covariates.

### Isolates and phenotypic characteristics

One-hundred-fifty-seven ESBL-*Ec* isolates (from 118 colonized travellers), detected at the first sampling after the trip, were analysed. The overall antimicrobial susceptibility results are shown in Table 3. Notably, only one isolate resistant to carbapenems was found. The sensitivity rate to ciprofloxacin was lowest in India (47.6%, 95% CI 37.1-58.2), whereas the lowest sensitivities to trimethoprim-sulfamethoxazon were in Sri Lanka (42.1%, 95% CI 23.1-63.7). Resistance rates against the other antibiotics tested were similar in all countries.

**Table 3:** **Prevalence of ESBL-** ***Ec*** **sensitive to additional antibiotics for each country visited** ^**a**^

	All countries (n = 157)	India (n = 82)	Bhutan (n = 15)	Nepal (n = 41)	Sri Lanka (n = 19)
AMC	58.6	59.8	73.3	56.1	47.4
PIP-TAZ	96.8	95.1	100.0	95.1	100.0
CEP	35.0	46.3	26.7	24.4	15.8
MEM	100.0	100.0	100.0	100.0	100.0
SXT	51.0	48.8	73.3	51.2	42.1
CIP	59.2	47.6	86.7	70.7	63.2
NF	98.1	98.8	93.3	97.6	100.0
FOS	99.4	100.0	100.0	97.6	100.0
AMK	95.5	96.3	100.0	95.1	89.5
TOB	82.2	76.8	100.0	87.8	79.0
COL	100.0	100.0	100.0	100.0	100.0

**Note:** AMC, amoxicillin-clavulanate; PIP-TAZ, piperacillin-tazobactam; CEP, cefepime; MEM, meropenem; SXT, thrimetoprim-sulfamethoxazole; CIP, ciprofloxacin; NF, nitrofurantoin; FOS, fosfomycin; AMK, amikacin; TOB, tobramycin; COL, colistin.

^**a**^stated as %.

### Molecular characteristics of the ESBL-*Ec*

**Figure 1:**
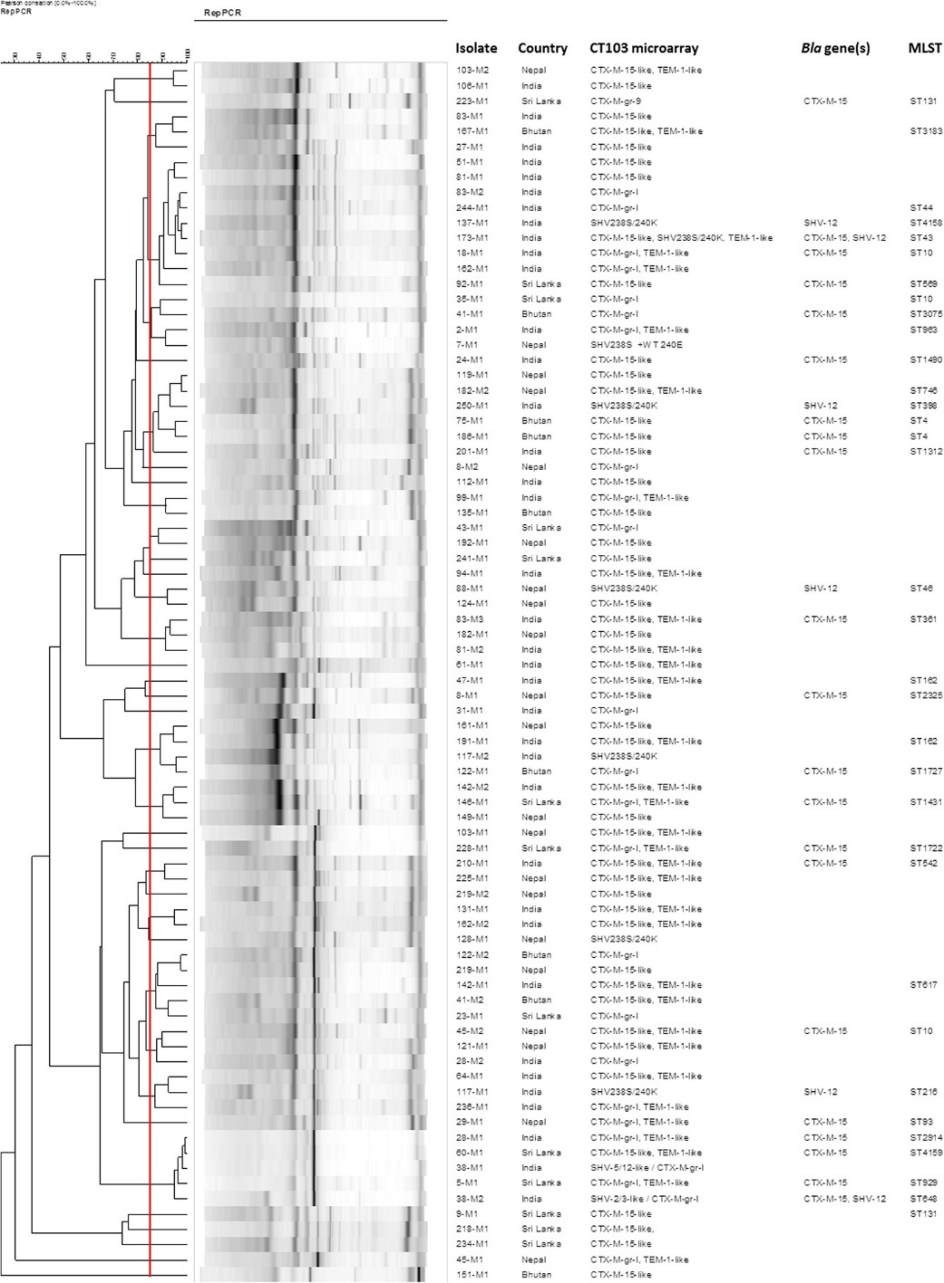
**Molecular characteristics of the 80 representative ESBL-producing isolates according to rep-PCR and multi locus sequencing.**

As shown in Figure 1, rep-PCR indicated that most of the *E. coli* isolates found in this study were sporadic and not clonally related. Only three pandemic strains (i.e., ST131 and ST648) were found and the predominant ESBL was the CTX-M-15. Neither a country-specific *E. coli* strain nor a country-specific ESBL was observed.

## Discussion

Colonization and infections with ESBL-*Ent* have been described to be associated with recent travelling [3, 8–13, 18–20, 26]. Depending on the region visited, colonization rates of over 70% have been recorded [8, 9, 11, 12]. In the current study, the highest colonization rates with ESBL-*Ec* were found in travellers returning from India, Nepal, and Bhutan. The figures are similar to those previously described in studies investigating asymptomatic travellers [8, 9, 11, 12], but considerably higher than the ones recorded in a recent Spanish study investigating travellers’ diarrhoea after returning from tropical and subtropical areas [13]. For a summary of existing prospective studies see Table 4.

**Table 4:** **Prospective studies on travel-associated colonization with ESBL-producing** ***Enterobacteriaceae*** **– rates and risk factors**

	Travellers (n) overall	Colonization rate (%) overall	Travellers (n) India/Indian subcontinent	Colonization rate (%) India/Indian subcontinent	Risk factors*
Current study	170	69.4	68	86.8	Travel Destination
					Length of Stay
					Visiting Friends and Relatives
					Consumption of Ice Cream & Pastry
Tängden et al. [8]	100	24.0	8	88.0	Travelling to India
					Gastroenteritis during Trip
Kennedy et al. [9]^a^	102	21.6	14	57.1	Gastroenteritis during Trip
					Antibiotics while Travelling
Weisenberg et al. [10]	28	25.0	7^b^	28.6	not done
Paltansing et al. [11]	370	30.5	25^c^	73.0^c^	Travelling to South and East Asia
Östholm-Balkhed et al. [12]	226	30.0	14	71.4	Travelling to Indian subcontinent, Asia, Africa north of equator
					Age
					Gastroenteritis during Trip

*Risk factors for becoming colonized for all the countries included in the respective study.

^a^risk factor for colonization with *E. coli* resistant to gentamicin, ciprofloxacin and/or third-generation cephalosporins.

^b^South Asia (not specified).

^c^South Asia: Bangladesh, India, Maldives, Nepal and Sri Lanka.

The rates observed in travellers returning from India, Nepal, and Bhutan, are in contrast to the significantly lower rate in travellers to the neighbouring country Sri Lanka. The reasons for these differences remain unclear. Differences in local prevalence are the most probable reason, but respective data is almost non-existent. Studies looking at clinical isolates from India showed rates of ESBL-*Ec* of 50-70% [27, 28]. In Nepal, 13.5% of *E. coli* and 16.5% of *K. pneumoniae* from urinary isolates were found to be ESBL producers [29]. In Sri Lanka, 52.1% of *E. coli* from clinical isolates were ESBL producers [30]. Regarding colonization with ESBL-*Ec*, one study from India found a rate of 15.4% in pregnant women [31]. However, no comparable data on carriage in the community for the other countries exists.

In this study, participants who visited friends and relatives showed a higher risk for becoming colonized than participants travelling as tourists only. As human-to-human transmission of ESBL-*Ent* is recorded [25], this observation supports the possible association between local prevalence and the risk for becoming colonized while travelling.

Similarly, a difference in the local occurrence of ESBL-*Ent* in the environment, in animals, and in food products might explain the different colonization rates in travellers. However, while studies on ESBL-*Ent* in the environment do exist [32], data for South Asia is scant. Studies on NDM-producing *Enterobacteriaceae* exist for India but there are no similar studies for the other countries [21]. Nevertheless, the fact that the *E. coli* strains found in this study are mostly of low-virulence STs (e.g., ST10 and ST10 complex) indicating a possible link with food, food-producing animals, and/or environment and not primarily “human” strains (e.g., ST131 and ST648) supports the hypothesis of an environmental origin of the colonization [33, 34].

Besides travel destination, consumption of ice cream and pastry was associated with becoming colonized in this study. Transmission of ESBL-*Ent* via the food chain has been described [6, 7], and as both ice cream and pastry contain animal products, such an association is plausible. However, ice cream, as well as pastry, can easily be contaminated during preparation or storage as they both are not heated again before consumption.

According to our analysis, drinking tap water is not associated with becoming colonized. This is completely contradictory to other studies which have found NDM-1-producing bacteria in the drinking water of New Delhi [21]. However, a previous study by Kennedy et al. has shown a negative association between drinking tap water and becoming colonized with resistant *E. coli* as well [9]. As in our study, no clear explanation for this seemingly counterintuitive result was found. Possibly, this observation is explained by drinking tap-water being a proxy for a certain way of travelling which protects against becoming colonized (i.e. hotels with chemically treated water). The fact that neither sleeping place nor eating place was associated with an increased risk for colonization could be explained by a lack of statistical power.

Increased length of stay as a risk factor for becoming colonized is self-explanatory. In contrast to previous studies [8, 9, 12, 18], suffering from gastrointestinal symptoms while travelling was not a risk factor for colonization. Antibiotic use while travelling, previously found to be associated with becoming colonized [9], was only reported by 7 travellers in our study.

By focusing on the Indian subcontinent, risk factors not previously described could be identified. However, given the colonization rate of almost 90% in travellers returning from India, the relevance of single risk factors is impossible to dissect. The search for risk factors in such a highly-endemic setting seems futile because colonization most likely originates from ubiquitous sources. This is supported by the fact that the *E. coli* isolates determined in the rep-PCR were mainly sporadic and less clonally related and that the MLST showed non-pandemic STs. The lack of clonal relatedness in ESBL-*Ec* acquired while travelling further supports the idea of various origins.

When looking at the high colonization rates with ESBL-*Ent* found in this study, the question about carbapenem-resistance arises for two reasons. Firstly, as a clear association between symptomatic infections with ESBL-*Ent* and recent travelling has been recorded [33], knowledge of high colonization rates in travellers might lead to an increase in the use of carbapenems, facilitating resistance development. More so, as almost 50% of the ESBL*-Ec* in this study were resistant or had intermediate resistance against trimethoprim-sulfamethoxazole or ciprofloxacin, mirroring the high additional resistance rates found in other studies [8, 11, 12, 19, 20]. Secondly, South Asia not only has high rates of ESBL-*Ent* but serves as an reservoir for NDM-1-*Ent*[16] as well. To date, colonization and infection with Carb-*Ent* occurs mainly in travellers with exposure to healthcare facilities [16] but has been described sporadically in healthy travellers as well [35]. Moreover, with NDM-1 already occurring in the environment [21], it might only be a question of time until Carb-*Ent* will be imported in significant numbers to low-endemicity regions through travellers as well.

Our study has several strengths and limitations. By focusing on the Indian sub-continent, we were able to identify new potential risk factors associated with becoming colonized. Additionally, by performing a molecular characterization of the detected strains, more information on the epidemiology of ESBL-producing *Enterobacteriaceae* in the respective countries could be gained. However, by only looking at a limited number of travel destinations, the results obtained are not generalizable to other travel destinations. On a technical level, colonization with multiple strains might not always have been detected and colonization below detection level cannot be excluded.

## Conclusions

Looking at the high colonization rates in travellers to the Indian subcontinent and the microbiological findings of mostly sporadic strains, the source of colonization most likely is ubiquitous (e.g. environment, food). Therefore, avoidance of colonization while travelling seems impossible, at least in highly-endemic areas. As a consequence, travel-related spread from high to low-endemicity areas will probably further increase in the future as the local prevalence in many regions of the world changes. As a clinical consequence, recent travel history of patients showing signs of infection should be taken into account when deciding on an empirical antibiotic treatment. Moreover, in the future, the travel-related spread of carbapenemase-producing *Enterobacteriaceae* needs to be closely monitored in order to detect when colonization with Carb-*Ent* in travellers starts to increase substantially.

## Authors’ original submitted files for images

Below are the links to the authors’ original submitted files for images. Authors’ original file for figure 1

## Abbreviations

ESBL: Extended-spectrum ß-lactamase
ESBL-Ent: Extended-spectrum ß-lactamase-producing *Enterobacteriaceae*
ESBL-Ec: Extended-spectrum ß-lactamase-producing *Escherichia coli*
Carb-Ent: Carbapenemase-producing *Enterobacteriaceae*
NDM-1: New Delhi metallo-beta-lactamase-1
MDR: Multidrug-resistant
MLST: Multi locus sequence typing
CI: Confidence interval
SD: Standard deviation
IQR: Inter quartile range
OR: Odds ratio
PPI: Proton pump inhibitor
VFR: Visiting friends and relatives
AMC: Amoxicillin-clavulanate
PIP-TAZ: Piperacillin-tazobactam
CEP: Cefepime
MEM: Meropenem
SXT: Thrimetoprim-sulfamethoxazole
CIP: Ciprofloxacin
NF: Nitrofurantoin
FOS: Fosfomycin
AMK: Amikacin
TOB: Tobramycin
COL: Colistin.
