# Supplementary figures and images for: High colonization rates of extended-spectrum β-lactamase (ESBL)-producing Escherichia coliin Swiss Travellers to South Asia– a prospective observational multicentre cohort study looking at epidemiology, microbiology and risk factors
Source: BMC Infect Dis. 2014 Oct 1;14:528. doi: 10.1186/1471-2334-14-528 (PMC4262238; doi:10.1186/1471-2334-14-528)

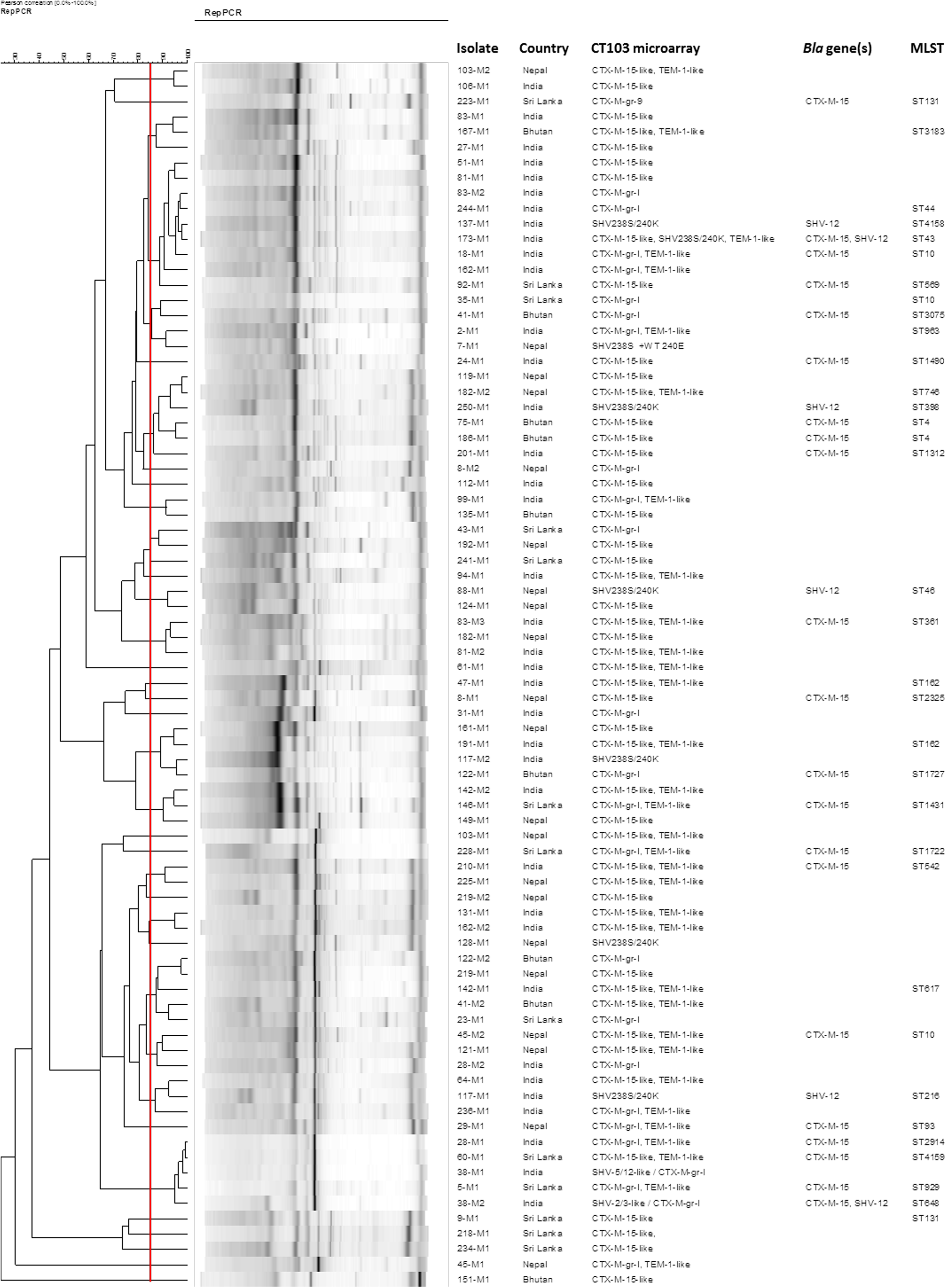

Supplement: Supplementary file 1 — Authors’ original file for figure 1 [file 12879_2014_3850_MOESM1_ESM.tif]
